# Characterization of soybean chitinase genes induced by rhizobacteria involved in the defense against *Fusarium oxysporum*

**DOI:** 10.3389/fpls.2024.1341181

**Published:** 2024-02-09

**Authors:** Jheng-Yan Chen, Hyunkyu Sang, Martin I. Chilvers, Chih-Hang Wu, Hao-Xun Chang

**Affiliations:** ^1^ Department of Plant Pathology and Microbiology, National Taiwan University, Taipei, Taiwan; ^2^ Department of Integrative Food, Bioscience and Biotechnology, Chonnam National University, Gwangju, Republic of Korea; ^3^ Department of Plant, Soil and Microbial Sciences, Michigan State University, East Lansing, MI, United States; ^4^ Institute of Plant and Microbial Biology, Academia Sinica, Taipei, Taiwan; ^5^ Master Program of Plant Medicine, National Taiwan University, Taipei, Taiwan; ^6^ Center of Biotechnology, National Taiwan University, Taipei, Taiwan

**Keywords:** *Bacillus amyloliquefaciens*, *Bradyrhizobium japonicum*, *Burkholderia ambifaria*, *Lysobacter enzymogenes*, *Pseudomonas fluorescens*, *Rhizobium rhizogenes*, *Glycine max*

## Abstract

Rhizobacteria are capable of inducing defense responses via the expression of pathogenesis-related proteins (PR-proteins) such as chitinases, and many studies have validated the functions of plant chitinases in defense responses. Soybean (*Glycine max*) is an economically important crop worldwide, but the functional validation of soybean chitinase in defense responses remains limited. In this study, genome-wide characterization of soybean chitinases was conducted, and the defense contribution of three chitinases (GmChi01, GmChi02, or GmChi16) was validated in *Arabidopsis* transgenic lines against the soil-borne pathogen *Fusarium oxysporum*. Compared to the *Arabidopsis* Col-0 and empty vector controls, the transgenic lines with GmChi02 or GmChi16 exhibited fewer chlorosis symptoms and wilting. While GmChi02 and GmChi16 enhanced defense to *F. oxysporum*, GmChi02 was the only one significantly induced by *Burkholderia ambifaria*. The observation indicated that plant chitinases may be induced by different rhizobacteria for defense responses. The survey of 37 soybean chitinase gene expressions in response to six rhizobacteria observed diverse inducibility, where only 10 genes were significantly upregulated by at least one rhizobacterium and 9 genes did not respond to any of the rhizobacteria. Motif analysis on soybean promoters further identified not only consensus but also rhizobacterium-specific transcription factor-binding sites for the inducible chitinase genes. Collectively, these results confirmed the involvement of GmChi02 and GmChi16 in defense enhancement and highlighted the diverse inducibility of 37 soybean chitinases encountering *F. oxysporum* and six rhizobacteria.

## Introduction

The composition of soil microbial communities varies depending on numerous factors, including the plant species or variety present. Plants release root exudates, which can attract beneficial microbes moving from bulk soil into the rhizosphere (Lugtenberg and Kamilova, 2009; Martinez-Medina et al., 2016; Lundberg and Teixeira, 2018). Many rhizobacteria, such as plant growth-promoting rhizobacteria (PGPR), can stimulate plant growth, and these rhizobacteria may enhance plant health by interacting directly or indirectly with soil-borne pathogens. The interactions can be generalized into three types: antagonism (Elnahal et al., 2022), parasitism (Tian et al., 2007), and induced systemic resistance (ISR) (Zhu et al., 2022). In terms of antagonism, rhizobacteria may secrete antibiotics or siderophores to antagonize or compete for nutrients with pathogens. Rhizobacteria such as *Bacillus amyloliquefaciens* and *Streptomyces* sp. exhibit these capabilities, and some strains have been developed into commercial products (Boubekri et al., 2022; Luo et al., 2022). Regarding parasitism, rhizobacteria may secrete enzymes such as chitinases that degrade fungal cell walls. Rhizobacteria such as *Burkholderia ambifaria* and *Enterobacter* sp. also possess predatory behaviors on fungi, thereby reducing fungal pathogens in the rhizosphere (Mousa et al., 2016; Stopnisek et al., 2016; Chang et al., 2021). Furthermore, due to the presence of microbe-associated molecular patterns (MAMPs) in most rhizobacteria, such as *Pseudomonas fluorescens* (Orozco-Mosqueda et al., 2023), pattern-triggered immunity (PTI) and ISR can be activated in the absence of soil-borne pathogens, leading to a phenomenon known as defense priming (Mauch-Mani et al., 2017; Salwan et al., 2023). Accordingly, the diverse mechanisms and interactions between plants and the rhizosphere microbes together contribute to the overall plant health and support agricultural sustainability.

The expression of pathogenesis-related protein (PR-protein) genes is one of the important responses in defense responses to combat pathogens, and many PR-protein genes have been confirmed to enhance defense responses in various mechanisms (Huang et al., 2016; Su et al., 2016; Luo et al., 2023; Wang Y et al., 2021). Among 17 families of PR-proteins (van Loon et al., 2006), the PR-3, PR-4, PR-8, and PR-11 proteins all encode plant chitinases, which contain the glycosyl hydrolases (GH) domain capable of breaking the β-1,4-glycosidic linkages of chitin, leading to disruption of fungal cell walls (Vaghela et al., 2022). These chitinases can be classified into GH18 and GH19 based on the similarity of their catalytic domains (CatD) (Kawase et al., 2004; Funkhouser and Aronson, 2007). The GH18 chitinases exhibit a barrel-like structure consisting of eight α-helices and eight β-sheets (Yang et al., 2010), and the GH19 chitinases possess a lysozyme-like domain composed of several α-helices (Kezuka et al., 2006). Plant chitinases have been further grouped into five classes (classes I–V) according to characteristics such as N-terminal sequences. While classes III and V belong to GH18, classes I, II, and IV belong to GH19 (Grover, 2012). Class I chitinases possess a chitin-binding domain (CBD) in their N-terminal region (Tang et al., 2004), and the C-terminal region of class I chitinases contains seven extended amino acids that facilitate their targeting to vacuolar and intracellular transport (Vaghela et al., 2022). Class II chitinases lack CBD in the N-terminal, and they are typically acidic proteins induced by pathogen infection and secreted to the extracellular space (Patil et al., 2000). Class III chitinases exhibit lysozyme activity without sequence similarity to classes I and II chitinases (Xu et al., 2016; Ma et al., 2017). Class IV chitinases possess both CBD and CatD similar to class I; however, due to deletions in the CBD and CatD domains, class IV chitinases are usually smaller than class I (Xu et al., 2016). Class V chitinases have a C-terminal extension for vacuolar targeting and may contain CBD (Taira et al., 2009; Grover, 2012). Accordingly, plant chitinases have evolved with diverse domains and variations.

The importance of plant chitinases in defense responses has been studied in several cases. For example, it was shown that 11 chitinase genes of rice were upregulated by *R. solani* infection. These rice chitinases were secreted into extracellular spaces, resulting in the degradation of fungal cell walls (Richa et al., 2016). Overexpression of the rice chitinase gene LOC_Os03g30470 enhanced defense against *Botrytis cinerea* and *Diplocarpon rosae* (Marchant et al., 1998; Núñez de Cáceres González et al., 2015). Overexpression of another rice chitinase gene, LOC_Os05g33130, increased defense responses to many diseases, and many studies have also demonstrated the defense contribution of various plant chitinases in different plant systems (**Table 1**). Other than the overexpression approach, gene silencing of the chili pepper chitinase gene CaChiIII7 resulted in larger foliar symptoms, less ROS accumulation in leaves, and reduced expression of defense-related genes (Ali et al., 2020). Collectively, the importance of plant chitinases in defense responses has been confirmed through overexpression and silencing approaches in different plant systems.

**Table 1:** Literature review of functionally characterized plant chitinases against fungal diseases.

Donor species	Gene ID	CAZy family	Class	Annotation	Recipient species	Targeting fungus	Ref.
Balsam pear	DQ407723.1 ABD66068.1	19	I	Mcchit1	Rice	*Magnaporthe grisea* *Rhizoctonia solani*	Li et al. (2009)
					Cotton	*Verticillium dahliae*	Xiao et al. (2007)
Barley	AJ276226.1 CAB99486.1	19	II	Chi2	Potato	*Alternaria solani*	Khan et al. (2017)
	AAA56786.1	19	II	CHI	Blackgram	*Corynespora cassiicola*	Chopra and Saini (2014)
					Tobacco	*Rhizoctonia solani*	Jach et al. (1995)
	M62904.1 AAA32941.1	19	II	Chi26	Wheat	*Fusarium graminearum*	Shin et al. (2008)
						*Puccinia recondite* *Puccinia striiformis* f. sp. *tritici* *Blumeria graminis*	Eissa et al. (2017)
	AAD28730.1	19	VII	Chi194	Tomato	*Fusarium oxysporum* f. sp. *lycopersici*	Girhepuje and Shinde (2011)
	KC899774.1 AGS38341.1	19	II	CEMB-chiII	Sugarcane	*Colletotrichum falcatum*	Tariq et al. (2018)
Bean	S43926.1 AAB23263.1	–	–	Chi CH5B	Cotton	*Verticillium dahliae*	Tohidfar et al. (2005)
					Canola	*Rhizoctonia solani*	Benhamou et al. (1993)
					Tobacco	*Rhizoctonia solani*	Brogue et al. (1991)
					Strawberry	*Botrytis cinerea*	Vellicce et al. (2006)
Chinese wild strawberry	MN709779 QLY89005.1	18	V	FnCHIT2	*Arabidopsis*	*Colletotrichum higginsianum*	Wen et al. (2020)
Cocoa	U30324 AAA80656.1	19	I	TcChi1	Cocoa	*Colletotrichum gloeosporioides*	Maximova et al. (2006)
Cucumber	NM_001308904.2 NP_001295833.1	18	III	CHI2	Cucumber	*Botrytis cinerea*	Kishimoto et al. (2004)
Eucommia ulmoides	KJ413009.1 AHX74093.1	19	I	EuCHIT2	Tobacco	*Erysiphe cichoracearum*	Dong et al. (2017)
Hanfu apple	LOC103401024 NP_001280823.1	19	II	MdCHI1	GL-3 apple	*Colletotrichum gloeosporioides* *Alternaria alternata*	Wang F et al. (2021)
Indian mustard	EF586206 ABQ57389.1	19	IV	Bj chitinase IV	Indian mustard	*Alternaria brassica*	Mir et al. (2021)
Mulberry	EXB55192.1	19	IV	MnChi18	*Arabidopsis*	*Botrytis cinerea*	Xin et al. (2022)
Maize	MG017374.1 AYK28286.1	19	I	Chit2	Maize	*Fusarium graminearum*	Dowd et al. (2018)
Pepper	KJ649334.1 AJF11981.1	19	IV	CaChitIV	*Arabidopsis*	*Hyaloperonospora arabidopsidis*	Kim et al. (2015)
Rice	LOC_Os03g30470 XP_015629397.1	–	–	RCH10	Rose	*Diplocarpon rosae*	Marchant et al. (1998)
					Lily	*Botrytis cinerea*	Núñez de Cáceres González et al. (2015)
	LOC_Os05g33130 XP_015640432.1	19	I	Chitinase2 Cht-2 RCC2 RCG3 RC7 ChtBD1 RC24	Banana	*Mycosphaerella fijiensis*	Kovács et al. (2013)
					Chrysanthemum	*Botrytis cinerea*	Takatsu et al. (1999)
					Cucumber	*Botrytis cinerea*	Tabei et al. (1998)
					Cucumber	*Botrytis cinerea*	Kishimoto et al. (2002)
					Grape	*Uncinula necator*	Yamamoto et al. (2000)
					Italian ryegrass	*Puccinia coronata*	Takahashi et al. (2005)
					Indica rice	*Rhizoctonia solani*	Datta et al. (2001)
					Peanut	*Cercospora arachidicola*	Iqbal et al. (2012)
					Rice	*Magnaporthe grisea*	Nishizawa et al. (1999)
					Strawberry	*Sphaerotheca humuli*	Asao et al. (1997)
					Tomato	*Alternaria solani* *Fusarium oxysporum* f. sp. *lycopersici*	Jabeen et al. (2015)
					Wheat	*Puccinia striiformis* f. sp. *tritici*	Huang et al. (2013)
	X54367.1 CAA38249.1	19	I	Chil1 RCC11 RChit	Finger millet	*Pyricularia grisea*	Ignacimuthu and Ceasar (2012)
					Grapevine	*Uncinula necator*	Nirala et al. (2010)
					Litchi	*Phomopsis* sp.	Das and Rahman (2018)
					Peanut	*Aspergillus flavus*	Prasad et al. (2013)
					Rice	*Rhizoctonia solani*	Rajesh et al. (2016)
	LOC_Os11g47510 ABA95474.1	18	–	–	Rice	*Rhizoctonia solani*	Richa et al. (2017)
Round-leaved sundew	KU516826.1 AMM76171.1	19	I	DrChit	Tobacco	*Trichoderma viride*	Durechova et al. (2019)
Strawberry	OQ211094.1 WGF83129.1	19	II	FvChi-14	*Arabidopsis*	*Colletotrichum higginsianum*	He et al. (2023)
Sweet potato	MN971588.1 QOD94995.1	19	II	IbChiA	Sweet potato	*Ceratocystis fimbriata*	Liu et al. (2020)
Sugar beet	A23392.1 CAA01677.1	19	IV	Chitinase IV	Silver birch	*Melampsoridium betulinum*	Pasonen et al. (2004)
						*Pyrenopeziza betulicola*	Pappinen et al. (2002)
Tobacco	X16938.1 CAA34812.1	19	I	Tob CHI	Tobacco	*Rhizoctonia solani*	Vierheilig et al. (1993)
					Peanut	*Cercospora arachidicola*	Rohini and Sankara Rao (2001)
Wild rice	EU850802.1 ACJ24349.1	19	IV	OgChitIVa	*Arabidopsis*	*Botrytis cinerea*	Pak et al. (2009)
Wild tomato	LOC107008831 XP_015063508.1	–	–	pcht28	Strawberry	*Verticillium dahliae*	Chalavi et al. (2003)
					Tomato	*Verticillium dahliae* race 2	Tabaeizadeh et al. (1999)
Zoysiagrass	–	19	II	Zjchi2	Zoysiagrass	*Rhizoctonia solani* AG2-2	Kang et al. (2017)

The advancement of high-throughput sequencing technology in the past decade has completed about 800 plant genomes (Marks et al., 2021; Sun et al., 2022), which speeded up the genome-wide characterization of plant chitinases in apple, *Arabidopsis thaliana*, *Brassica rapa*, cotton, cucumber, mulberry, rice, and tea (Grover, 2012; Xu et al., 2016; Chen et al., 2018; Bartholomew et al., 2019; Mir et al., 2020; Bordoloi et al., 2021; Haxim et al., 2022; Xin et al., 2022). For example, seven of the 24 chitinase genes discovered in the *A. thaliana* genome, such as AT1G19810, AT2G43570, AT2G43580, AT2G4359, and AT3G47540, were found to be upregulated upon infection by *B. cinerea* and *Pseudomonas syringae*. In the case of rice, 49 chitinase genes were characterized, and transcriptome analysis identified Os01g18400, Os01g19750, Os10g28050, and Os11g47510 being upregulated in response to *Magnaporthe grisea* infection (Grover, 2012). In the genomes of *B. rapa* and tea, 33 and 49 chitinase genes were discovered, respectively. Upregulation of several chitinase genes was also found during infections of the clubroot pathogen and three tea pathogens (Chen et al., 2018; Bordoloi et al., 2021). These findings demonstrate the power of high-throughput sequencing in genome-wide identification of plant chitinases, which also enable investigations for their expressions to different microbes.

Soybean (*Glycine max*) is one of the most important crops worldwide, and soybean diseases have been one of the major yield-limiting stresses for decades (Bandara et al., 2020; Bradley et al., 2021; Allen et al., 2022). However, there were limited studies on the functional validation of soybean chitinases (Lv et al., 2022) and their expressions induced by rhizobacteria. Therefore, this study performed a genome- and transcriptome-wide identification of soybean chitinases induced by *B. ambifaria* and validated the potential of soybean chitinases in defense against *Fusarium oxysporum*. In addition, transcriptomic analyses were conducted to profile soybean chitinases induced by six rhizobacteria, including *B. amyloliquefaciens*, *Bradyrhizobium japonicum*, *B. ambifaria*, *Lysobacter enzymogenes*, *P. fluorescens*, and *Rhizobium rhizogenes* (previously known as *Agrobacterium rhizogenes*). The study not only completed a comprehensive identification and validation of soybean chitinases induced by rhizobacteria but also highlighted the regulatory consensus and diversity among soybean chitinases to different rhizobacteria.

## Results

### Genome-wide identification of soybean chitinase genes

**Figure 1:**
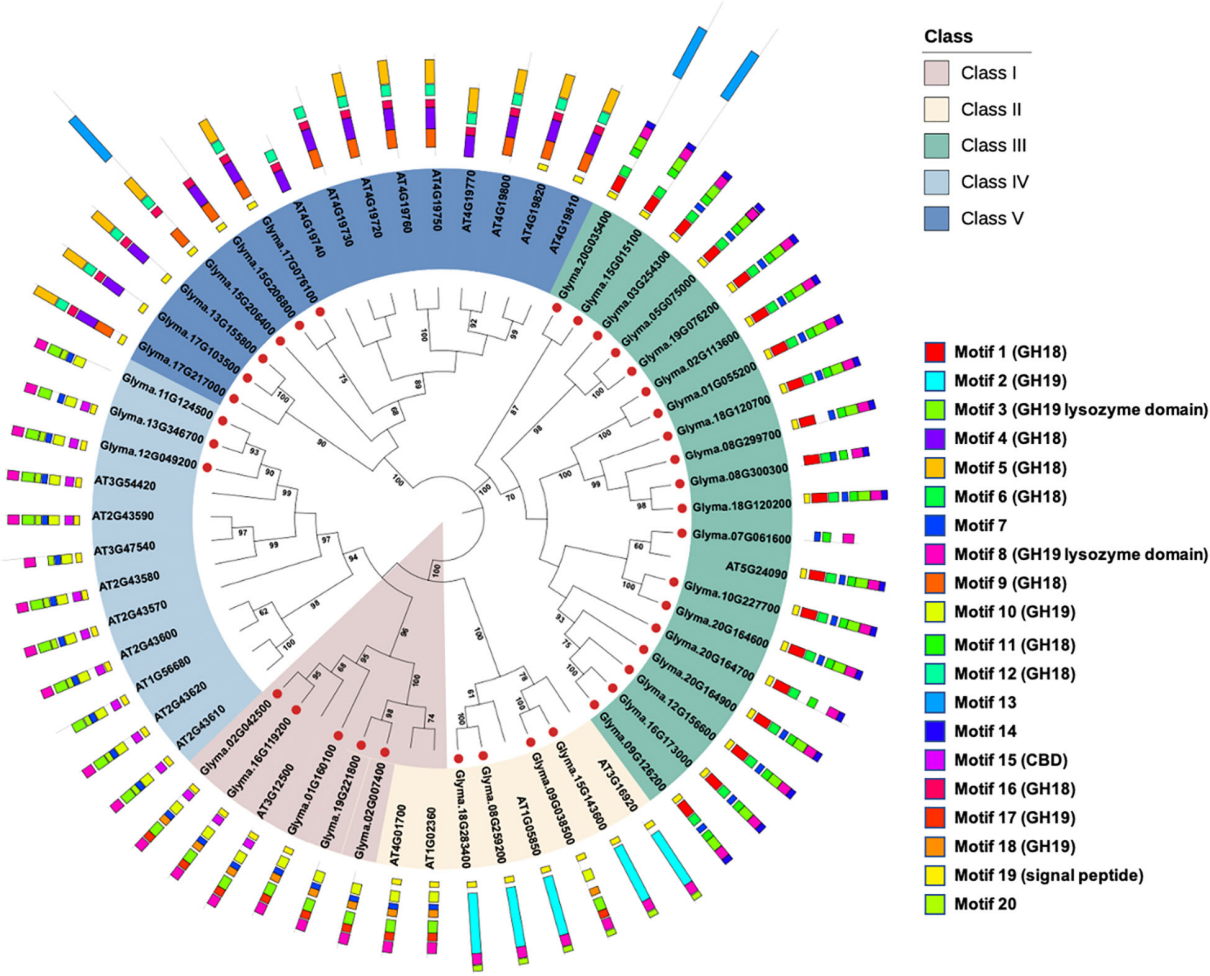
Genome-wide identification of soybean chitinase genes. The conserved HMM domains of the glycosyl hydrolase (GH) GH18 (PF00704) and GH19 (PF00182) chitinases were used as templates to identify soybean chitinases in the soybean reference genome. The 37 putative chitinases were further grouped using the neighbor-joining method into five classes according to the classification system built on *Arabidopsis thaliana* chitinases. There were 5, 4, 19, 3, and 6 chitinase genes in classes I, II, III, IV, and V, respectively, and there were 20 conserved motifs detected in the soybean chitinases. Bootstrap values above 60 were shown in the nodes.

A search of the GH18 (PF00704) and GH19 (PF00182) domains identified 37 chitinase genes in the soybean genome, and all of them were predicted with an N-terminal signal peptide. Following the classification system of *Arabidopsis* chitinase genes, the soybean chitinase genes can be further divided into five genes in class I, four genes in class II, nineteen genes in class III, three genes in class IV, and six genes in class V (**Figure 1**). Using MEME analyses for characterizing motifs, the conserved GH18 motifs were identified in classes III and V, and the GH19 motifs were identified in classes I, II, and IV. As reported in the previous literature (Ma et al., 2017), class III chitinases harbor both GH18 motifs and GH19 lysozyme domain (motifs 3 and 8) as a classification signature. These results together confirmed that the HMMER method in genome-wide identification of soybean chitinase genes is robust and precise.

One controversial classification appeared for Glyma.01G160100, Glyma.02G007400, and Glyma.19G221800, which should be classified as class II for the absence of CBD (motif 15) if they followed the conventional classification for *Arabidopsis* chitinase genes (Patil et al., 2000). Although the presence or absence of CBD has been used to identify class I or IV chitinases (Patil et al., 2000; Grover, 2012; Xu et al., 2016), the MEME analyses found exceptions not only in soybean but also in *Arabidopsis*. For example, AT3G47540 was recognized as class IV chitinase because CBD was absent. In addition, although AT1G02360 and AT4G01700 were grouped as class II chitinases based on the absence of CBD, both phylogenetic and MEME analyses suggested that their sequences and motif structures were closer to the class I chitinases. Therefore, this study suggests a phylogeny-based classification for soybean chitinase genes (**Figure 1**), where classes II, III, and V chitinase genes do not contain CBD motif, while classes I and IV chitinase genes may contain CBD motif. According to such criteria, Glyma.01G160100, Glyma.02G007400, and Glyma.19G221800 were classified as class I chitinases.

### Gene expression of soybean chitinases in a tritrophic RNA-Seq experiment

**Figure 2:**
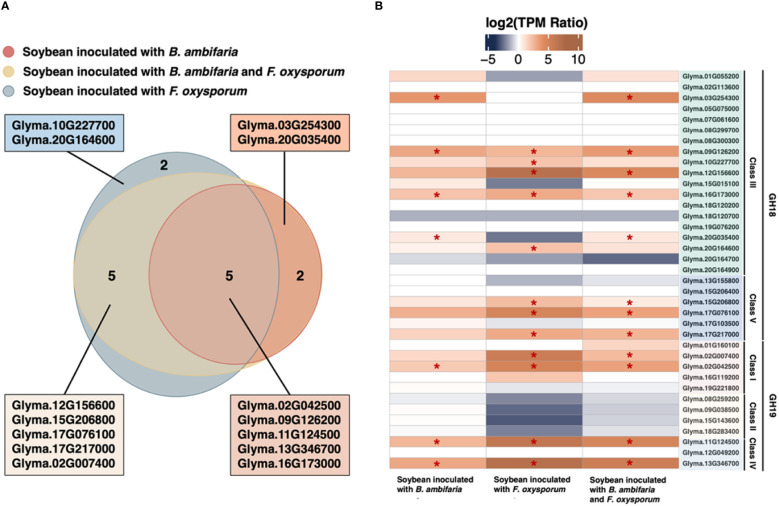
Soybean chitinase genes in response to the inoculation of *Burkholderia ambifaria*, *Fusarium oxysporum*, and co-inoculation. **(A)** Venn diagram suggests 12 chitinases being upregulated in response to *F*. *oxysporum* inoculation, while 7 chitinase genes were significantly induced by *B. ambifaria* in the absence of *F*. *oxysporum*. There were 5 consensus responding to the inoculation of *B. ambifaria*, *F*. *oxysporum*, and co-inoculation. **(B)** Gene expression of the 37 soybean chitinases, and the asterisk indicated the statistical significance of differential expression at a *q*-value of 0.05.

Among the soybean chitinase genes, 12 genes exhibited upregulation in response to inoculation with *F. oxysporum* (**Figure 2A**). The top 5 upregulated genes included Glyma.13G346700, Glyma.12G156600, Glyma.11G124500, Glyma.02G007400, and Glyma.02G024500, which displayed a log_2_ fold change of 7.27, 7.23, 6.44, 5.89, and 4.97, respectively. Meanwhile, three among these five genes (Glyma.13G346700, Glyma.11G124500, and Glyma.02G024500) were also upregulated by the inoculation of rhizobacterium *B. ambifaria* in the absence of *F. oxysporum* (**Table 2**). In addition, Glyma.16G173000 and Glyma.09G126200 were also induced by *B. ambifaria*, but the upregulation of these two genes by *F. oxysporum* was not as high as the others. These results highlighted that these five soybean chitinase genes (Glyma.02G024500, Glyma.09G126200, Glyma.11G124500, Glyma.13G346700, and Glyma.16G173000) participated in the defense responses induced by *B. ambifaria*, and three of the five genes were listed in the top 5 important chitinase genes in the defense responses to *F. oxysporum* infection (**Figure 2B**). Indeed, upon the co-inoculation of *F. oxysporum* and *B. ambifaria*, for which the biomass of *F. oxysporum* was reduced by the antagonism of *B. ambifaria* (Chang et al., 2021), the upregulation of Glyma.13G346700, Glyma.11G124500, and Glyma.02G024500 was about 20% to 33% reduced compared to inoculation with *F. oxysporum* alone (**Table 2**). Collectively, Glyma.13G346700, Glyma.11G124500, and Glyma.02G024500 became the research focus not only for their inducibility but also for their expression trends reflecting the biotic stress created by the inoculation of *F. oxysporum*.

**Table 2:** Soybean chitinase genes induced by the inoculation of *Fusarium oxysporum*, *Burkholderia ambifaria*, and co-inoculation.

Gene ID	*F. oxysporum*		*B. ambifaria*		Co-inoculation	
	Log_2_FC	*q*-value	Log_2_FC	*q*-value	Log_2_FC	*q*-value
**Glyma.02G007400**	**5.89**	**3.59E−18**	**n.s.**	**n.s.**	**2.64**	**4.28E−04**
**Glyma.02G042500**	**4.97**	**1.15E−50**	**1.87**	**2.17E−04**	**3.32**	**1.86E−22**
Glyma.03G254300	n.s.	n.s.	3.13	3.30E−02	3.92	9.04E−04
Glyma.09G126200	2.57	7.85E−08	3.12	5.88E−51	3.57	7.64E−91
Glyma.10G227700	2.08	6.45E−05	n.s.	n.s.	n.s.	n.s.
**Glyma.11G124500**	**6.44**	**8.66E−125**	**2.69**	**3.39E−06**	**5.07**	**4.81E−73**
**Glyma.12G156600**	**7.23**	**1.73E−22**	**n.s.**	**n.s.**	**4.82**	**4.41E−04**
**Glyma.13G346700**	**7.27**	**9.46E−69**	**3.28**	**1.12E−17**	**5.77**	**9.76E−69**
Glyma.15G206800	2.35	1.73E−25	n.s.	n.s.	0.71	5.09E−05
Glyma.16G173000	3.00	2.42E−05	2.12	8.11E−06	1.99	7.85E−05
Glyma.17G076100	4.88	1.36E−08	n.s.	n.s.	3.12	5.07E−04
Glyma.17G217000	3.09	1.46E−06	n.s.	n.s.	2.72	2.23E−04
Glyma.20G035400	n.s.	n.s.	0.88	8.63E−03	0.98	3.90E−04
Glyma.20G164600	2.43	1.96E−02	n.s.	n.s.	n.s.	n.s.

n.s., genes that were not being significantly upregulated. Bold values are the top 5-upregulated chitinase genes in response to *F. oxysporum*

### Phylogenetic analysis for soybean chitinases in defense responses

**Figure 3:**
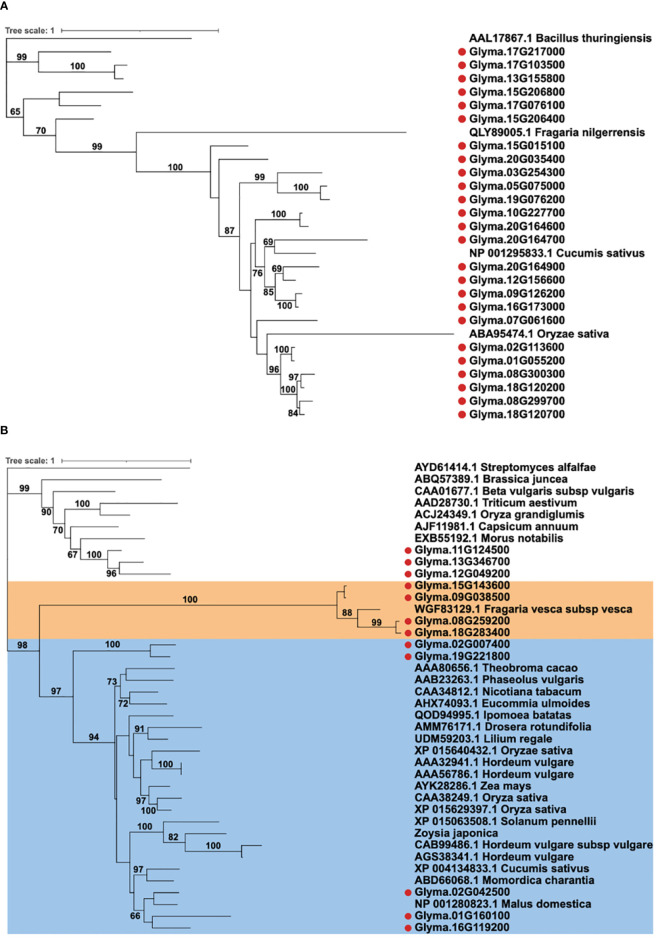
Phylogenetic analysis of soybean chitinase genes with functionally validated plant chitinases. **(A)** Soybean GH18 chitinases were analyzed with 3 functionally validated chitinases from Chinese wild strawberries, cucumber, and rice. **(B)** Soybean GH19 chitinases were analyzed with 27 functionally validated chitinases from multiple plant species. Soybean chitinase GmChi02, GmChi01, and GmChi16 (Glyma.02G042500, Glyma.01G160100, and Glyma.16G119200) are phylogenetically close to each other and grouped with most functionally validated plant chitinases. Unlike GmChi02, GmChi01 and GmChi16 were not upregulated by *F*. *oxysporum* nor induced by *B. ambifaria*. Bootstrap values above 60 in the Maximum likelihood tree are shown.

In order to assess the potentials of Glyma.13G346700, Glyma.11G124500, and Glyma.02G024500 in defense responses, a phylogenetic analysis for the 37 soybean chitinase genes was performed together with functionally validated plant chitinases from the literature (**Table 1**). For GH 18 chitinases, there were only three functionally validated plant chitinases, including cucumber, Chinese wild strawberry, and rice that can enhance defense responses to *B. cinerea*, *Colletotrichum higginsianum*, and *R. solani*, respectively (Kishimoto et al., 2004; Richa et al., 2017; Wen et al., 2020) (**Figure 3A**). There were more studies that confirmed the function of GH19 plant chitinases, and 27 functionally validated plant chitinases from apple, barley, cocoa, common bean, cucumber, maize, pepper, rice, and wheat were included in the phylogenetic analysis together with 12 GH19 soybean chitinases. These 12 soybean genes can be categorized into three groups (**Figure 3B**). The first group contains three soybean chitinase genes (Glyma.11G124500, Glyma.12G049200, and Glyma.13G346700), and the closest ortholog gene is the pepper CaChitIV, which enhances defense responses to *Arabidopsis* downy mildew (Kim et al., 2015). The second group includes four soybean chitinase genes, but only a strawberry gene, FvChi-14, which enhances *Arabidopsis* defense responses to *C. higginsianum*, is phylogenetically neighboring to these four genes (He et al., 2023). The last group with Glyma.01G160100, Glyma.02G024500, and Glyma.16G119200 clustered with apple, barley, bitter melon, cucumber, wild tomato, and zoysiagrass that were previously shown to contribute to the defense responses against multiple fungal pathogens, including many soil-borne fungi such as *F. oxysporum*, *R. solani*, and *Verticillium dahliae* (**Table 1**). In addition, two chitinase genes (Glyma.02G007400 and Glyma19G221800) were in the same clade but located distantly from the three soybean chitinase genes abovementioned. Among these genes, Glyma.02G024500 is the one that responded to the inoculation of *F. oxysporum* and *B. ambifaria*. On the other hand, the phylogenetically closed Glyma.01G160100 and Glyma.16G119200 did not seem to participate in the defense responses at least to *F. oxysporum*, nor be induced by *B. ambifaria*. The observation raised a question whether Glyma.01G160100, Glyma.02G024500, and Glyma.16G119200 (hereafter referred to a GmChi01, GmChi02, and GmChi16) all contain antifungal capability or if only Glyma.02G024500 remains antifungal. Accordingly, GmChi01, GmChi02, and GmChi16 were selected for functional validation.

### Functional validation for soybean chitinases in defense responses

**Figure 4:**
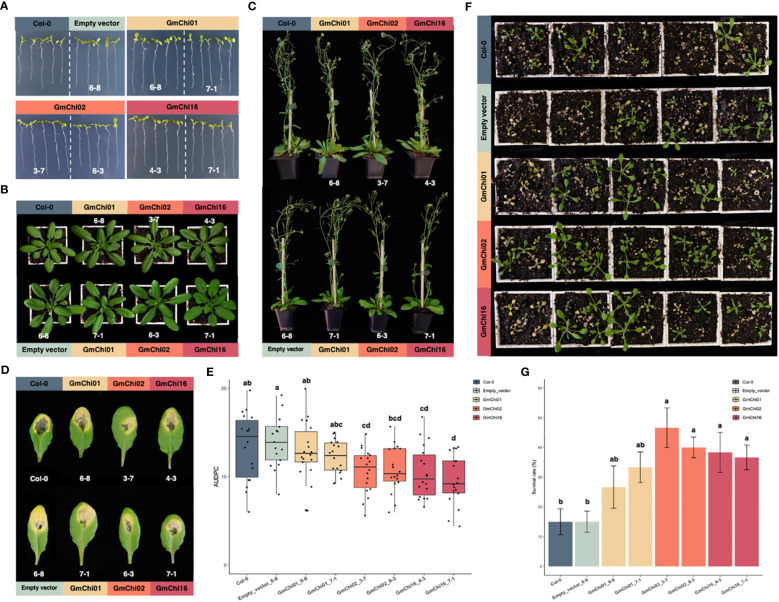
Phenotypes of *Arabidopsis* transgenic lines with GmChi01, GmChi02, or GmChi16. **(A)** Seedling root length, **(B)** Rosette leaves, and **(C)** Plant height of *Arabidopsis* transgenic lines exhibited no significant difference to the wild type Col-0 or *Arabidopsis* transgenic line with empty vector. **(D)** *Arabidopsis* transgenic lines with GmChi02 or GmChi16 exhibited mild symptoms after the inoculation of *F. oxysporum*. **(E)** Quantification of foliar symptoms after the inoculation of *F. oxysporum*. **(F)** *Arabidopsis* transgenic lines with GmChi02 or GmChi16 exhibited better seedling survival rates after the inoculation of *F. oxysporum*. **(G)** Quantification of seedling survival rates after the inoculation of *F. oxysporum*. Different letters indicate significant difference among the *Arabidopsis* transgenic lines (P< 0.05).

The homozygous transgenic *Arabidopsis* lines overexpressing empty vector (EV), GmChi01, GmChi02, or GmChi16, respectively, were validated for the expression of soybean chitinases (**Supplementary Figure S1**) before using their T_4_ generation for experiments. For seedling root length, rosette leaves, and plant height, the transgenic lines (EV_6-8, GmChi01_6-8, GmChi01_7-1, GmChi02_3-7, GmChi02_6-3, GmChi16_4-3, and GmChi16_7-1) exhibited no difference in phenotypes (**Figures 4A–C**). However, the area under the disease progress curve (AUDPC) of these lines inoculated with *F. oxysporum* exhibited significant differences. The AUDPC of transgenic *Arabidopsis* overexpressing GmChi02 and GmChi16 were significantly lower than the controls (**Figures 4D, E**). Although transgenic *Arabidopsis* overexpressing GmChi01 exhibited reduced AUDPC in appearance, statistical analysis did not detect a significant difference. Meanwhile, identical results can be observed in soil inoculation with the conidial suspension of *F. oxysporum*, where transgenic *Arabidopsis* overexpressing GmChi02 and GmChi16 exhibited less seedling wilt. Transgenic *Arabidopsis* overexpressing GmChi01 again showed better survival in appearance, but the statistical analysis did not detect any significance (**Figures 4F**, **G**). These results indicate that GmChi02 and GmChi16 were indeed phylogenetically and functionally close, and these two soybean chitinases enhanced defense responses to *F. oxysporum* infection. However, the gene regulation of GmChi01, GmChi02, and GmChi16 appeared to be diversified, and only GmChi02 exhibited inducibility in response to *B. ambifaria*.

### Gene preference induced by different rhizobacteria on soybean chitinases

**Figure 5:**
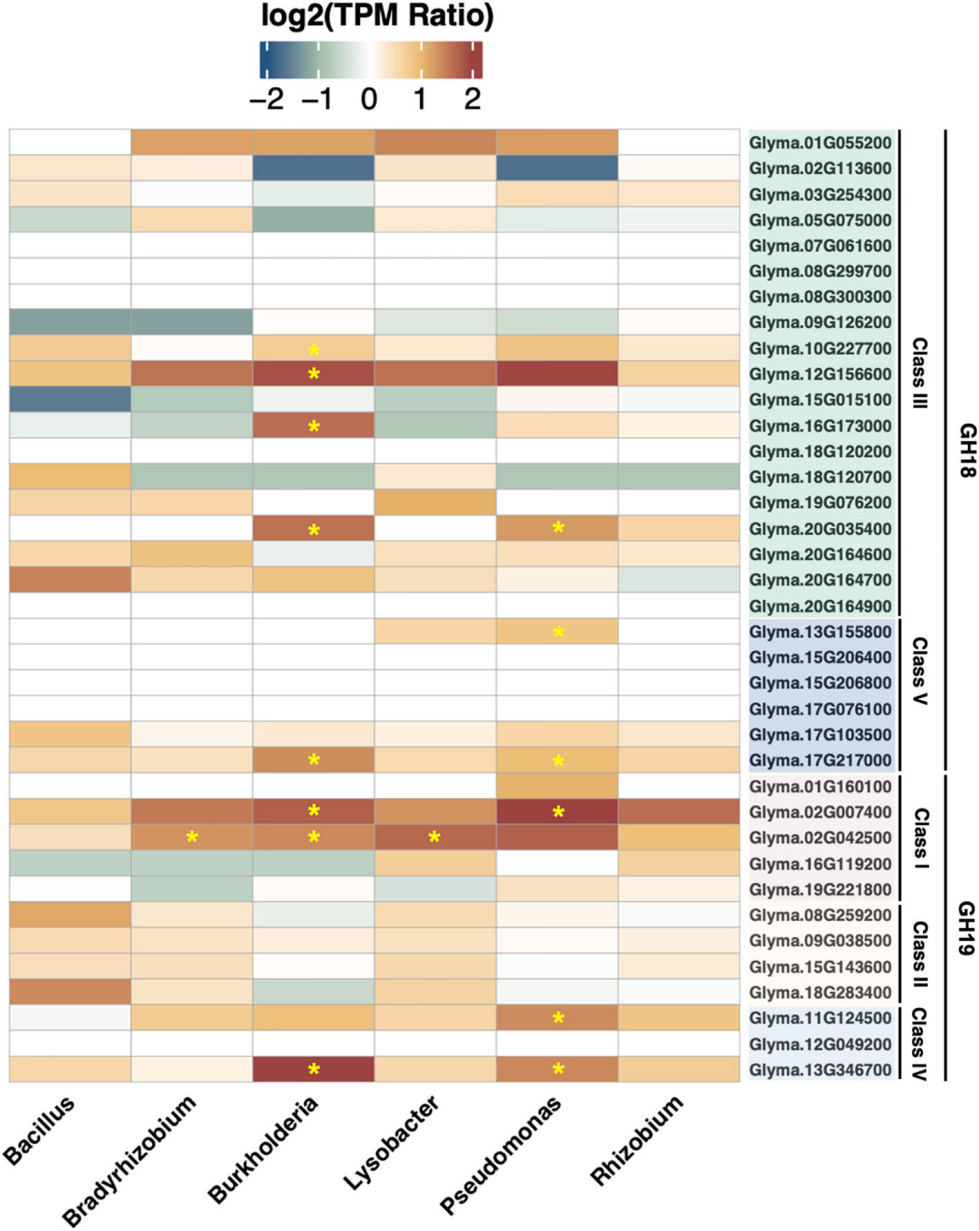
Gene expression of soybean chitinases in response to six rhizobacteria. Asterisks highlight the significant upregulation induced by the inoculation of each rhizobacterium. GmChi02 responded to all rhizobacteria, and the inoculation of *B. japonicum*, *B. ambifaria*, and *L. enzymogenes* reached statistical significance. GmChi01 only responded to *P. fluorescens* without statistical significance, and GmChi16 was upregulated by *L. enzymogenes* and *R. rhizogenes* without statistical significance.

In order to survey the inducibility of soybean chitinase genes, six rhizobacteria from different genera were applied to soybean taproot to characterize gene expressions. As a result, *Bacillus amyloliquefaciens*, *Bradyrhizobium japonicum*, *B. ambifaria*, *Lysobacter enzymogenes*, *Pseudomonas fluorescens*, and *Rhizobium rhizogenes* upregulated zero, one, eight, one, six, and zero chitinase genes, respectively. Although there were some chitinase genes showing upregulation based on the average log_2_ fold change, variation within biological replicates may reduce the confidence in detecting statistical significance for cases such as GmChi02 (Glyma.02G024500) in response to *P. fluorescens*. Nonetheless, the survey confirmed that soybean chitinase genes responded differently to various rhizobacteria, where the expression of 10 chitinase genes (Glyma.02G007400, Glyma.02G042500, Glyma.10G227700, Glyma.11G124500, Glyma.12G156600, Glyma.13G155800, Glyma.13G346700, Glyma.16G173000, Glyma.17G217000, and Glyma.20G035400) were significantly induced by at least one rhizobacterium, and the expression of nine chitinase genes (Glyma.07G061600, Glyma.08G299700, Glyma.08G300300, Glyma.12G049200, Glyma.15G206400, Glyma.15G206800, Glyma.17G076100, Glyma.18G120200, and Glyma.20G164900) remain unchanged to all rhizobacteria (**Figure 5**).

Specifically, GmChi02 can be significantly induced by *B. diazoefficiens*, *B. ambifaria*, and *L. enzymogenes*. On the other hand, GmChi01 or GmChi16 did not reach statistical significance for any rhizobacteria. As for other chitinase genes such as Glyma.13G346700, Glyma.12G156600, Glyma.11G124500, and Glyma.02G007400 that were induced by *F. oxysporum* infection (**Figure 2B**), Glyma.13G346700 and Glyma.02G007400 can be significantly induced by *B. ambifaria* and *P. fluorescens*. On the other hand, Glyma.12G156600 was upregulated by *B. ambifaria*, while Glyma.11G124500 was upregulated by *P. fluorescens*. These results suggest that soybean chitinase genes upregulated in the defense responses to *F. oxysporum* infection all interacted with at least one of the six rhizobacteria. Therefore, transcription factor-binding sites may have emerged during the co-evolution between soybeans and these rhizobacteria.

### Identification of transcription factor and transcription factor-binding sites for the rhizobacteria-inducible soybean chitinase genes

**Figure 6:**
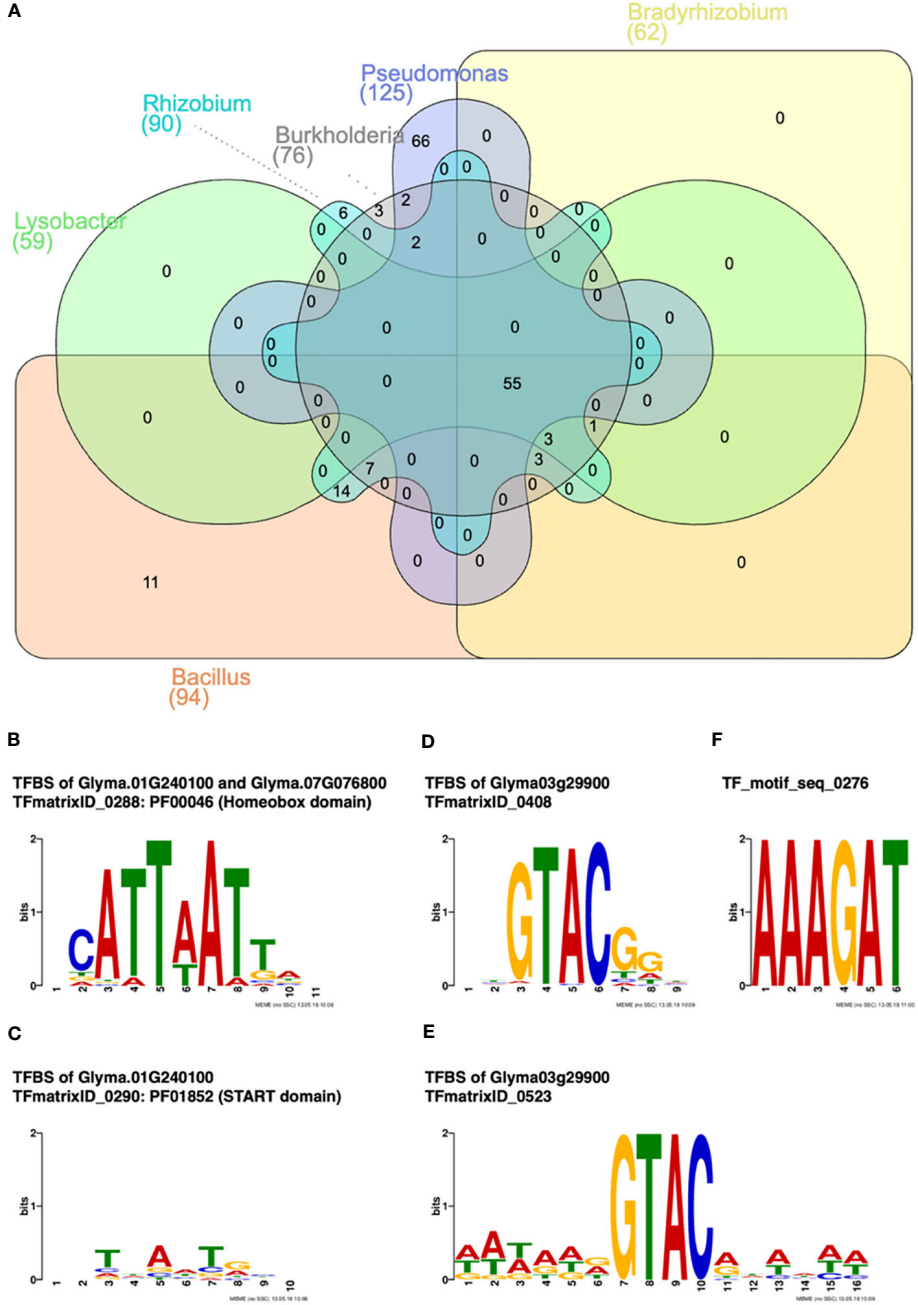
Transcription factor (TF) and TF-binding site (TFBS) analysis for rhizobacteria-inducible soybean chitinase genes. **(A)** Venn diagram of inducible soybean chitinase genes by *Bacillus amyloliquefaciens*, *Bradyrhizobium japonicum*, *Burkholderia ambifaria*, *Lysobacter enzymogenes*, *Pseudomonas fluorescens*, and *Rhizobium rhizogenes*. Only three soybean TFs responded to *B. ambifaria*, specifically. **(B)** Two TFs containing the homeobox domain in response to the inoculation of *B. ambifaria*, specifically. **(C)** One TF containing the START domain in response to the inoculation of *B. ambifaria*, specifically. **(D, E)** One TF containing the SQUAMOSA promoter-binding (SPB) domain in response to the inoculation of *B. ambifaria*, specifically. **(F)** The nodule-specific *cis*-regulatory motif was found in response to *B. amyloliquefaciens*, *B. japonicum*, *B. ambifaria*, *L. enzymogenes*, and *R. rhizogenes*.

In order to identify the potential regulatory motifs, the 5′ UTR and 3′ UTR of soybean chitinase genes that responded to the six rhizobacteria were analyzed. There were 94, 62, 76, 59, 125, and 90 soybean transcription factor-binding sites (TFBSs) associated with transcription factors (TFs) for soybean chitinase genes induced by *B. amyloliquefaciens*, *B. japonicum*, *B. ambifaria*, *L. enzymogenes*, *P. fluorescens*, and *R. rhizogenes*, respectively (**Figure 6A**). Among these genes, there were 55 TFBSs associated with TFs consensually identified for all rhizobacteria, while there were only 11, zero, three, zero, 66, and six TFs exhibiting a specificity to *B. amyloliquefaciens*, *B. japonicum*, *B. ambifaria*, *L. enzymogenes*, *P. fluorescens*, and *R. rhizogenes*, respectively. Focusing on the TF enriched for interacting with *B. ambifaria*, there were three unique TFs, including two homeobox domain TFs (Glyma.01G240100 and Glyma.07G076800) and one SQUAMOSA promoter-binding protein (SBP)-box TF (Glyma03g29900) (**Figures 6B–E**).

As for TFBS without associated TFs, only one *cis*-regulatory element, NODCON1GM1, was found for *B. amyloliquefaciens*, *B. japonicum*, *B. ambifaria*, *L. enzymogenes*, and *R. rhizogenes* in contrast to the inducible and noninducible chitinase genes (**Figure 6F**). Based on the consensus and diversified inducibility of soybean chitinase genes, the results indicated that the regulatory mechanism of chitinase genes may have co-evolved with soybean–rhizobacteria interaction.

## Discussion

The benefits of rhizobacteria for plant health have been greatly recognized in different aspects. Other than direct antagonism against soil-borne pathogens, rhizobacteria may stimulate defense responses to provide sustainable plant protection. However, some scholars have pointed out that crops grown in fields may already be in a constant defense priming and/or induced systemic resistance (ISR) state because they are persistently interacting with rhizobacteria, and simply applying or exposure to rhizobacteria may not be sufficient to combat pathogens (Pieterse et al., 2014). Other literature indicates the density of rhizobacteria is a crucial key for defense priming, or ISR. For example, the minimum bacterial density required for *P. fluorescens* alone to induce ISR in laboratory conditions required 10^5^ (Raaijmakers, 1995), and it may need to be higher in field conditions. Therefore, it could be challenging to achieve a sufficient population density of rhizobacteria for defense priming or ISR throughout the entire growing season (Walters et al., 2013). An alternative strategy is molecular breeding for important defense genes to bypass the reliance on bacterial density. For example, Rushanaedy et al. (2012) found the koa tree chitinase genes AKchit1a and AKchit1b were significantly upregulated in the resistant cultivars against *F. oxysporum* compared to susceptible cultivars, providing a screening criterion for disease resistance. Another application of plant chitinase genes is the early detection of biotic stresses. For example, the chitinase activity of papaya fruits was significantly increased upon *Colletotrichum gloeosporioides* infection during both the preharvest and storage stages. Since *C. gloeosporioides* is a pathogen exhibiting a latent infection stage, the expression of chitinase genes may serve as diagnostic biomarkers for asymptomatic fruits (Lucas-Bautista et al., 2020). Regardless of being selected as breeding targets or diagnosis biomarkers, the characterization of plant chitinase genes can provide novel insights and a comprehensive understanding of defense responses for a plant species.

This study performed genome- and transcriptome-wide identifications of soybean chitinases and functionally validated three phylogenetically close-related genes (GmChi01, GmChi02, and GmChi16) for their involvement in defense responses. The results showed that GmChi02 and GmChi16 enhanced defense responses to *F. oxysporum*, but only GmChi02 can be induced by *B. ambifaria*. In the transcriptomic characterization of GmChi02 in different rhizobacteria, the results confirmed a significant upregulation by *B. ambifaria* and *P. fluorescens*, and the expression of GmChi02 also responded to the inoculation of *B. amyloliquefaciens*, *B. japonicum*, *L. enzymogenes*, as well as *R. rhizogenes*. The observation indicates that GmChi02 may have co-evolved with multiple rhizobacteria to induce defense responses against soil-borne pathogens. On the other hand, although GmChi16 exhibited an equivalent defense effect as GmChi02, the expression of GmChi16 only responded to the inoculation of *L. enzymogenes* and *R. rhizogenes*. Collectively, these observations indicate that the regulatory mechanism of soybean chitinase genes may have diversity not only in the coding sequence level for functionality but also in the expression level in terms of inducibility.

Several TFs and TFBSs have been shown to regulate plant chitinase expression. For example, the homeodomain leucine zipper III TF CsHB15 of cucumber was found to bind the promoter of CsChi23 and induce gene expression in response to *F. oxysporum* (Bartholomew et al., 2022). The R2R3-MYB TF of brown mustard was shown to recognize the W-box-like-4 (Wbl-4) element to activate BjCHI1 in response to *B. cinerea* (Gao et al., 2016). Another example is the LrWRKY2 of lily, which induced LrCHI2 expression in response to *F. oxysporum* (Li et al., 2021). However, whether plant chitinase genes harbor conserved TF and TFBS in response to rhizobacteria has not been assessed. In this study, several TF genes and motifs were highlighted by contrasting the TFs and TFBS motifs between the inducible and non-inducible soybean chitinase genes in each rhizobacterium (**Supplementary Table S2**). One with particular interest would be the NODCON1GM, which has been known to be a nodule-specific regulatory element (Wang et al., 2022). Mutation and deletion of NODCON1GM (5′-AAAGAT) or another regulatory element, NODCON2GM (5′-CTCTT), were shown to decrease the number of nodule formations (Jørgensen et al., 1991). The presence of NODCON1GM in the promoters of rhizobacteria-inducible chitinase genes suggests a possibility that the regulatory mechanism to drive chitinase genes may rely on a similar manner as the regulatory element NODCON1GM. Additional studies on the emergence of NODCON1GM in some but not all soybean chitinase genes, along with studies on the presence of NODCON1GM in the promoters of other soybean PR-protein genes, may further illuminate the evolution of rhizobacteria-induced defense responses.

Research has shown that soybean has diverse rhizobacteria, including *Bradyrhizobium*, *Bacillus*, *Burkholderia*, and *Rhizobium* species (Biate et al., 2014; Zhong et al., 2019; Han et al., 2020; Yamazaki et al., 2021), that could affect soybean yield and disease incidence (Chang et al., 2017; Hussain et al., 2018). Future studies may focus on the selection pressure derived from soybean rhizobacteria on the expressions of PR-protein genes and the regulatory mechanisms of defense responses induced by different rhizobacteria. These research advances may provide a broad knowledge of the application of beneficial rhizobacteria to enhance plant health.

## Materials and methods

### Plant and microbial materials

For routine cultivation of *Arabidopsis thaliana*, the seeds were surface-sterilized using 70% ethanol and 50% Clorox bleach (Oakland, CA, USA). After rinsing five times with sterile water, the seeds were placed in the dark at 4°C for 48 h for vernalization. Subsequently, the seeds were planted in a soil mixture (peat moss:vermiculite:perlite = 6:1:1) and cultured in a long-day condition (16 h light/8 h dark) at 22°C.

For routine growth of rhizobacteria, *Bradyrhizobium japonicum* USDA6 (BCRC 80814^T^) was cultured in yeast mannitol broth (0.2 g/L K_2_HPO_4_, 0.2 g/L MgSO_4_·7H_2_O, 10.0 g/L mannitol, 0.05 g/L NaCl, 0.3 g/L yeast extract; pH 6.2). The other bacterial species, including *Bacillus amyloliquefaciens* ATCC23350 (BCRC 11601^T^), *Burkholderia ambifaria* AMMD ATCCBAA-244 (NRRL B-23395^T^), *Lysobacter enzymogenes* ATCC29487 (BCRC 11654^T^), *Pseudomonas fluorescens* ATCC 13525 (BCRC 11028^T^), and *Rhizobium rhizogenes* K599 (Lifeasible, Shirley, NY 11967, USA) were cultured in Nutrient Broth (HiMedia, Mumbai, India). All rhizobacteria were cultured at 28°C with 125 rpm shaking. To establish the correlation between optical density (OD) 600 and colony-forming units (CFU), bacterial suspensions at OD600 value of 0.5 were diluted and quantified on plates, and linear regression was applied in the later experiment for estimating CFU of bacterial suspensions.

For routine growth of *Fusarium oxysporum* f.sp. *rapae* (BCRC FU31513), the fungus was subcultured on potato dextrose agar (PDA) at 28°C without light every 7 days. For producing conidia, the fungus was cultured in synthetic nutrient-poor broth (SNB) (0.5 g/L MgSO_4_·7H_2_O, 1 g/L KH_2_PO_4_, 1 g/L KNO_3_, 0.5 g/L KCl, 0.2 g/L glucose, and 0.2 g/L sucrose) (Moura et al., 2020) in the dark at 28°C and 125 rpm for 7 days. The conidia suspension was adjusted to a concentration of 1 × 10^6^ conidia/ml.

### Genome-wide identification and phylogenetic analysis of soybean chitinases

To identify chitinase genes in the soybean genome, the HMMs of the GH18 (PF00704) and GH19 (PF00182) protein domains were downloaded from the Pfam database (Mistry et al., 2021). Subsequently, HMMER v3.3.2 was applied to search PF00704 and PF00182 in the ‘Williams 82’ (W82) (Gmax_508_Wm82.a4.v1.protein) at a threshold of 1^−10^ *E*-value (Finn et al., 2011). The presence of GH18 or GH19 domain was double-checked using the NCBI Conserved Domain Database at a threshold of 1^−20^ *E*-value. In addition, protein tertiary structure was assessed by predicting the folded structure of each soybean chitinase gene protein sequence using ColabFold (Mirdita et al., 2022). Furthermore, MEME v5.4.1 was utilized at a setting of a maximum motif length of 300 and a number of motifs of 20 to identify conserved motifs within the protein sequences (Bailey and Elkan, 1994). The Protparam (Gasteiger et al., 2005), SignalP5.0 (Almagro Armenteros et al., 2019), and DeepLoc-1.0 (Almagro Armenteros et al., 2017) webtools were employed to investigate the amino acid composition, molecular weight, and isoelectric point of soybean chitinase proteins.

The protein sequences of soybean chitinases were aligned with 24 *Arabidopsis thaliana* chitinases sourced from the TAIR database (Lamesch et al., 2012). Alignment was performed using MAFFT v7 (Katoh et al., 2019), and the phylogenetic tree was constructed using the neighbor-joining (NJ) method in MEGA-X (Kumar et al., 2018). Additionally, the protein sequences of soybean chitinases were aligned with functionally validated chitinase sequences from 21 plant species (**Table 1**). The phylogenetic tree was constructed using the maximum likelihood (ML) method in IQ-TREE v2.2.0 (Nguyen et al., 2015). The visualization of the phylogenetic trees was generated using iTOL (Letunic and Bork, 2021).

### Transcriptomic analysis of soybean chitinases

The tritrophic RNA-Seq data were obtained from a previous study on the gene expression of *F. oxysporum* in the roots of the soybean variety ‘Jack’ under the influence of the antagonistic bacterium *B. ambifaria* (Chang et al., 2021). The data can be categorized into four treatments: (1) soybean roots without *B. ambifaria* or *F. oxysporum*, (2) soybean roots inoculated with *B. ambifaria*, (3) soybean roots inoculated with *F. oxysporum*, and (4) soybean roots simultaneously inoculated with both *B. ambifaria* and *F. oxysporum*. Each treatment consisted of three biological replicates, totaling 12 samples. The RNA-Seq was performed using the Illumina HiSeq 4000 platform (Illumina, San Diego, CA, USA). The raw data underwent quality control to keep reads with a Phred score ≥ 30 using the FASTQC and FASTX-ToolKit v0.0.14. The soybean W82 transcriptome (Gmax_508_Wm82.a4.v1.cds.fa) was used as a template for Kallisto v0.46.1 (Bray et al., 2016). Subsequently, differential gene expression analysis was conducted using the R package Sleuth v0.30 (Pimentel et al., 2017) at a threshold of 0.05 *q*-value. Transcript per million (TPM) measurements of the 37 soybean chitinase genes were presented in a heatmap using the R package ComplexHeatmap v2.13.1 (Gu et al., 2016).

In the RNA-Seq experiment of soybean root inoculated by six rhizobacteria, the W82 soybean seeds were sterilized in 1% bleach for 15 min, followed by five rinses with sterile water. The sterilized seeds were vernalized in sterile water at 28°C without light to better synchronize the germination rate. The next day, the seed coats were removed, and the seeds were placed on 1.5% water agar plates in a growth chamber at 28°C without light for 3 days. After the seeds germinated and the hypocotyls elongated to approximately 3 cm to 5 cm, the seedlings were transferred to new water agar (WA) plates, where 100 µl (approximately 1 × 10^7^ CFU/ml) of bacterial suspension was inoculated onto the soybean hypocotyls. The control group was inoculated with ddH_2_O.

The inoculated soybean seedlings were further incubated in a growth chamber at 28°C without light. After incubating for 2 days, the frozen taproot samples were homogenized in liquid nitrogen with Invitrogen™ TRIzol™ Reagent (Thermo Fisher Scientific, Waltham, MA, USA), followed by the extraction workflow using chloroform and isopropanol. With two biological replicates per rhizobacteria and control, a total of 14 samples were sent to RNA-Seq using the Illumina NovaSeq 6000 platform in a 150-bp pair-ended platform (Biotools, New Taipei City, Taiwan).

### Molecular cloning of GmChi01, GmChi02, and GmChi16

Three chitinase genes, namely Glyma.01G160100 (GmChi01), Glyma.02G042500 (GmChi02), and Glyma.16G119200 (GmChi16), were PCR amplified from the soybean W82 genomic DNA using primers with a SpeI site at the 3′ end (GmChi01_F_SpeI/GmChi01_R_SpeI; GmChi02_F_SpeI/GmChi02_R_SpeI; GmChi16_F_SpeI/GmChi16_R_SpeI) (**Supplementary Table S1**) via the Phusion^®^ High-Fidelity DNA Polymerase (New England Biolabs, Ipswich, MA, USA). The PCR sizes of three chitinase genes were 2227 bp (GmChi01), 2243 bp (GmChi02), and 1417 bp (GmChi16), and the amplicons were treated with SpeI before being cleaned up using the GenepHlowTW Gel/PCR Kit (Geneaid, New Taipei City, Taiwan). The T4 DNA Ligase (NEB) was used to ligate chitinase amplicons into the pCAMBIA1302 vector pretreated with shrimp alkaline phosphatase (rSAP) (NEB). The ligation mixture was heat-shock transformed into *Escherichia coli* DH5α competent cells (Yeastern Biotech, New Taipei City, Taiwan) and selected on kanamycin. Colony PCR was performed using specific primers for each chitinase gene (**Table 2**) using the SMB All-1 DNA Polymerase Premix (StarMoonBio, New Taipei City, Taiwan). The constructs (pCAMBIA1302::GmChi01, pCAMBIA1302::GmChi02, and pCAMBIA1302::GmChi16) were purified using the EasyPure Plasmid DNA Mini Kit (Bioman, New Taipei City, Taiwan) before being sent for Sanger sequencing (Genomics Co., New Taipei City, Taiwan).

### Generation of *Arabidopsis* transgenic lines using *Agrobacterium* floral dipping

The *Agrobacterium tumefaciens* GV3101 was cultured in the 523 liquid medium (8 g/L casein hydrolysate, 2 g/L K_2_HPO_4_, 0.3 g/L MgSO_4_·7H_2_O, 10 g/L sucrose, 4 g/L yeast extract; pH 6.9) supplemented with rifampicin (50 mg/L) and streptomycin (100 mg/L) at 125 rpm shaking for 24 h at 28°C. Upon the optical density (OD600) reaching 1.0 to 1.5, the *Agrobacterium* suspension was centrifuged at 4,500 rpm at 4°C. The bacterial pellet was resuspended in 20 mM CaCl_2_ as competent cells. Three soybean chitinase constructs and an empty vector were individually transformed into *A. tumefaciens* GV3101 using the freeze–thaw method, including a 30-s liquid nitrogen immersion and a 37°C water bath for 5 min. The transformed bacterial cells were selected by kanamycin (50 mg/L). Colony PCR, using gene-specific primer pairs, was used for validation. The *Agrobacterium* strains were stored in 523/Kan+/Rif+/Strep+ medium with 50% glycerol at −80°C.

For *Agrobacterium* floral dipping, the desired *Agrobacterium* strains were freshly prepared in the 523/Kan+/Rif+/Strep+ medium, and the bacterial pellets were resuspended in a 5% sucrose solution containing 0.02% Silwet L-77 (PhytoTech Lab, Lenexa, KS, USA) to OD600 = 0.6 as inoculum. The floral dipping procedure followed the protocol by Zhang et al. (2006) with slight modifications; in brief, the siliques and pollinated flowers were removed from 6-week-old *A. thaliana* ecotype Col-0, and the unopened *Arabidopsis* inflorescences were immersed in the *Agrobacterium* inoculum for 20 s. After immersion, the plants were kept in humid chambers before being routinely cultured at 22°C.

The *Arabidopsis* seeds harvested after floral dipping represented the T_1_ generation. The T_1_ seeds were selected on the MS medium containing 40 ppm hygromycin. The T_1_ plants with hygromycin resistance were further PCR-confirmed before generating the T_2_ seeds. The T_2_ seeds were selected on hygromycin to estimate the Mendelian segregation (3:1) for each T_1_ lineage. T_1_ lineages with a single T-DNA insertion were propagated into the T_3_ generation. Approximately 100 T_3_ seeds of each lineage were screened on hygromycin. If the T_3_ germination rate was approximately 100%, the lineage was considered to be homozygous. On the other hand, if the germination rate was around 75%, the lineage was considered to be heterozygous at the T_2_ generation. Phenotyping and pathogenicity assay were only performed using the progenies of homozygous T_2_ lineages (**Supplementary Figure S1**).

For the *Arabidopsis* transgenic lines, the expressions of soybean chitinase (GmChi01, GmChi02, or GmChi16) were confirmed via RT-qPCR. In brief, foliar RNA of transgenic lines was extracted by the TRIzol procedure described above. The raw RNA was treated with the TURBO DNase (Thermo Fisher Scientific) before cDNA synthesis using the SuperScript IV Reverse Transcriptase (Thermo Fisher Scientific) and oligo d(T)18 primer (Bioman). RT-qPCR was performed using the iQ™ SYBR^®^ Green Supermix (Bio-RAD, Hercules, CA, USA) with the primers (GmChi01_qPCR; GmChi02_qPCR; GmChi16_qPCR; AtACT7) (**Supplementary Table S1**) on the CFX ConnectTM Real-Time PCR Detection System (Bio-RAD). Three-step thermocycling conditions were set: initial denaturation at 95°C for 5 min, 40 cycles of denaturation at 95°C for 15 s, annealing at 62°C for 10 s, and extension at 72°C for 10 s. The gene expression was presented using the formula ΔCt = Ct_target gene_ − Ct_AtACT7_. The melty curve of each RT-qPCR amplicon was assessed to confirm specificity, and the amplification efficiencies of primers were optimized to ensure the use of 2^−ΔCt^ (Livak and Schmittgen, 2001).

### Phenotyping and pathogenicity assay on *Arabidopsis* transgenic lines

The hypocotyl length, radical length, rosette area, and stem length were measured for the wild-type *A. thaliana* Col-0 and the *Arabidopsis* transgenic lines. Hypocotyl and radical lengths were measured after 1 week of growth on MS medium, while rosette area was calculated using the software Easy Leaf Area (Easlon and Bloom, 2014) after another 3 weeks in pots. Stem length measurements were conducted at the 6-week growth stage. The experiments were repeated twice, and there were 15 biological replicates each time. These data were collected for statistical analyses.

The detached leaf assay was applied to evaluate the defense responses of *Arabidopsis* lines. A 5-mm-diameter PDA plug with the mycelial edge of *F. oxysporum* f.sp. *rapae* was inoculated onto *Arabidopsis* leaves with a needle wound on the leaf surface. The inoculated leaves were grown for 4 weeks. An ordinal disease index (DI) was measured daily for 1 week, for which the index at 0, 1, 2, 3, 4, and 5 indicates 0%, 1%–10%, 11%–25%, 25%–50%, 51%–75%, and 76%–100% of leaf yellowing, and index at 6 indicates a complete wilt and dead leaf (**Supplementary Figure S2**). The area under the disease progress curve (AUDPC) was calculated (Sparks et al., 2008). The experiments were repeated three times, and there were nine biological replicates each time.

In addition, soil inoculation was performed by spreading the conidial suspension of *F. oxysporum* f. sp. *rapae* onto the 1-week-old *Arabidopsis* lines. After inoculation, the pots were covered with plastic lids to maintain humidity and placed in the greenhouse at room temperature (25°C ± 2°C). The plastic lids were removed after 10 days postinoculation. The experiments were repeated three times, and there were four biological replicates each time. These data were collected for statistical analyses.

### Statistical analysis for phenotypic data

The R v4.0.5 environment and RStudio v1.4.17 were used for statistical analyses. All data were analyzed using the nonparametric Kruskal–Wallis rank sum test, and Dunn’s test was applied for mean separation at a threshold of *α* = 0.05.

### Identification of TF and TFBS for the rhizobacteria-inducible soybean chitinase genes

Soybean chitinase genes were grouped into two categories, including rhizobacteria-inducible chitinase genes (regardless of up- or downregulation) and nonrhizobacteria-inducible chitinase genes (**Supplementary Table S2**). The upstream 2,000 bp 5′UTR and downstream 500 bp 3′UTR of these genes were subjected to PlantPAN3.0 analysis (Chow et al., 2019) using soybean as the model plant for searching TF and TFBS at 90% frequency of support.

## Data availability statement

The RNA-Seq data were deposited in the NCBI BioProject PRJNA987518 and the analyses also included the previously published data in the NCBI BioProject PRJNA512928.

## Author contributions

J-YC: Conceptualization, Data curation, Formal analysis, Investigation, Methodology, Resources, Software, Validation, Visualization, Writing – original draft, Writing – review & editing. HS: Conceptualization, Investigation, Methodology, Writing – review & editing. MC: Conceptualization, Investigation, Methodology, Writing – review & editing. C-HW: Conceptualization, Investigation, Methodology, Writing – review & editing. H-XC: Conceptualization, Data curation, Funding acquisition, Investigation, Methodology, Project administration, Supervision, Visualization, Writing – original draft, Writing – review & editing.

## Supplementary material

Supplementary Figure 1 RT-PCR validation for the expression of GmChi01, GmChi02, and GmChi16 in the *Arabidopsis* transgenic lines.

Supplementary Figure 2 The disease index scale for detached leaf assay.

Supplementary Table 1 Primer sequences.

Supplementary Table 2 Soybean chitinase genes inducible or non-inducible by six rhizobacteria.
