# Supplementary material for: Characterization of soybean chitinase genes induced by rhizobacteria involved in the defense against Fusarium oxysporum
Source: Front Plant Sci. 2024 Feb 9;15:1341181. doi: 10.3389/fpls.2024.1341181 (PMC10884886; doi:10.3389/fpls.2024.1341181)
Supplement: Supplementary Figure 1 — RT-PCR validation for the expression of GmChi01, GmChi02, and GmChi16 in the Arabidopsis transgenic lines. [file DataSheet_1.pdf]

Supplementary Material

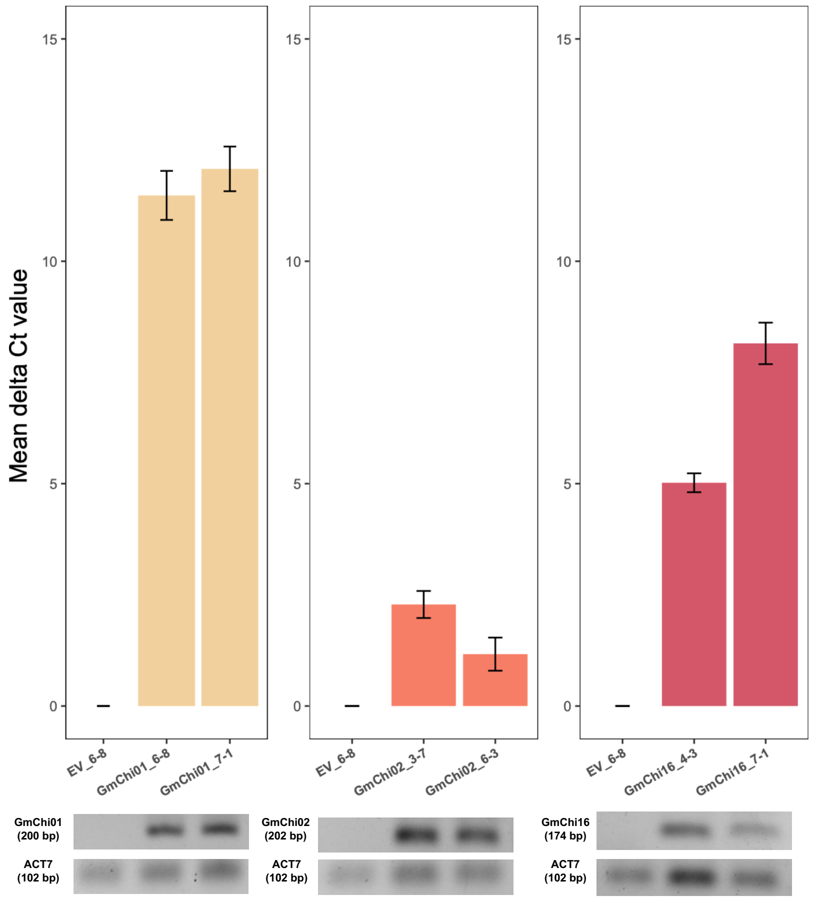

Fig. S1. RT-PCR validation for the expression of GmChi01, GmChi02, and GmChi16 in the *Arabidopsis* transgenic lines. The EV_6-8 line expresses empty vector was used as control.

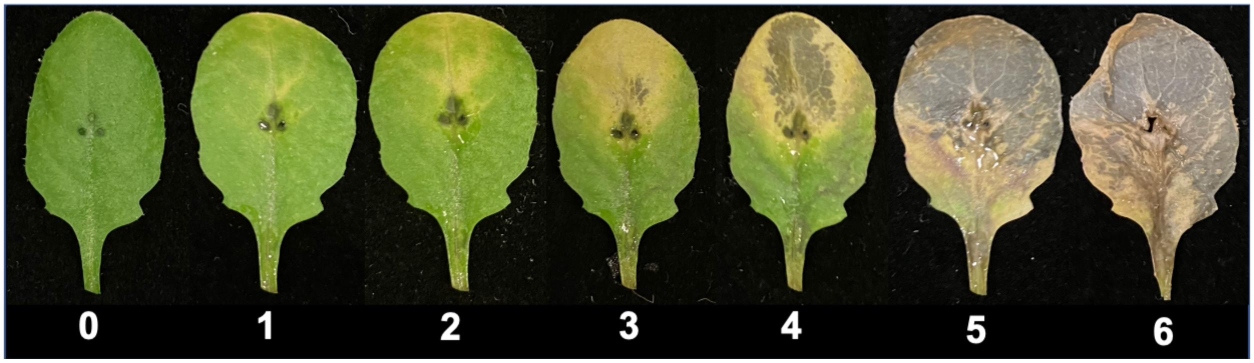

Fig. S2. The disease index scale for detached leaf assay. *Arabidopsis* leaves were inoculated with *Fusarium oxysporum* agar blocks, and the severity was rated from 0 to 6.

Table S1. Primer sequences

Primer name	Primer sequence (5'-3')	Application
GmChi01_F_SpeI	TCT C**AC TAG T**AT GAA ATT AGA CAC TAT CTT CGC C	Cloning
GmChi01_R_SpeI	TGT G**AC TAG T**TT AAT CTT GAT CAA AGG ACT TTT GGC	Cloning
GmChi02_F_SpeI	AAG G**AC TAG T**AT GAA AAA CAT GAA ATT GTG TTC GG	Cloning
GmChi02_R_SpeI	TGT G**AC TAG T**TT AAG CAA ATG GCC TTT GAT TGT TAC	Cloning
GmChi16_F_SpeI	CCT T**AC TAG T**AT GGG TAA CAT GAA ATT GTG TCC	Cloning
GmChi16_R_SpeI	TGT G**AC TAG T**TT AAA ATG GCC TTT GAT TGT TG	Cloning
GmChi01_83R	ACT CTG ATG AGG TTA CCA GC	Colony PCR
GmChi02_184R	TCC ACA GTA GGA ATC TGT GTC	Colony PCR
GmChi16_274R	AGA ACG GCT AAT GGT GTT GC	Colony PCR
pCAMBIA1302_F	CAC GAC ACA CTT GTC TAC TCC	Colony PCR
GmChi01_qPCR_F	ATT AGC AAG GCA CCA TGT GC	RT-qPCR
GmChi01_qPCR_R	GGC TTA TTG GCT TGT GAT GTC	RT-qPCR, Sanger sequencing
GmChi02_qPCR_F	CAC TCG CAA GAA GGA GAT TG	RT-qPCR
GmChi02_qPCR_R	TGT AAG TTG GAT TGG TCC TC	RT-qPCR, Sanger sequencing
GmChi16_qPCR_F	TAT CAA CGA ACG CAA CCA GG	RT-qPCR
GmChi16_qPCR_R	CTG GAT ACT AGA TCC GGG TC	RT-qPCR, Sanger sequencing
GmChi01_689R	ACT TAC TGT GTC AGC TGA AGG	Sanger sequencing
GmChi01_1350R	CGG TCC CAT GAC AAA GAG G	Sanger sequencing
GmChi02_467R	AAC CTG TGG TTT CAT GAG AAG	Sanger sequencing
GmChi02_1627R	AAG AGT GCA TAA AAA GTA GGG C	Sanger sequencing
GmChi16_469R	ATC AAT GAA CTA ACC TGT GG	Sanger sequencing
pCAMBIA1302_R	TGC CCA TTA ACA TCA CCA TC	Sanger sequencing
AtACT7_F	AGCACCTTCCAACAGATGTGGA	RT-qPCR
AtACT7_R	ATAAGACAAGACACACTTAGAAGCA	RT-qPCR
GmELF1B_F	GTTGAAAAGCCAGGGGACA	RT-qPCR
GmELF1B_R	CTTACCCCTTGAGCGTGG	RT-qPCR
GmUKN2_F	TGTGCTCTGTGAAGAGATTG	RT-qPCR
GmUKN2_R	TCATAATCTGTGTGCAGTTC	RT-qPCR

Table S2. Soybean chitinase genes inducible or non-inducible by six rhizobacteria

Bacillus amyloliquefaciens		***Bradhyrhizobium japonicum***		***Burkholderia ambifaria***	
Inducible	Non-inducible	Inducible	Non-inducible	Inducible	Non-inducible
Glyma.02G007400	Glyma.01G055200	Glyma.01G055200	Glyma.01G160100	Glyma.01G055200	Glyma.01G160100
Glyma.02G042500	Glyma.01G160100	Glyma.02G007400	Glyma.03G254300	Glyma.02G007400	Glyma.07G061600
Glyma.02G113600	Glyma.07G061600	Glyma.02G042500	Glyma.07G061600	Glyma.02G042500	Glyma.08G299700
Glyma.03G254300	Glyma.08G299700	Glyma.02G113600	Glyma.08G299700	Glyma.02G113600	Glyma.08G300300
Glyma.05G075000	Glyma.08G300300	Glyma.05G075000	Glyma.08G300300	Glyma.03G254300	Glyma.09G126200
Glyma.08G259200	Glyma.11G124500	Glyma.08G259200	Glyma.10G227700	Glyma.05G075000	Glyma.12G049200
Glyma.09G038500	Glyma.12G049200	Glyma.09G038500	Glyma.12G049200	Glyma.08G259200	Glyma.13G155800
Glyma.09G126200	Glyma.13G155800	Glyma.09G126200	Glyma.13G155800	Glyma.09G038500	Glyma.15G015100
Glyma.10G227700	Glyma.15G206400	Glyma.11G124500	Glyma.15G206400	Glyma.10G227700	Glyma.15G143600
Glyma.12G156600	Glyma.15G206800	Glyma.12G156600	Glyma.15G206800	Glyma.11G124500	Glyma.15G206400
Glyma.13G346700	Glyma.17G076100	Glyma.13G346700	Glyma.17G076100	Glyma.12G156600	Glyma.15G206800
Glyma.15G015100	Glyma.18G120200	Glyma.15G015100	Glyma.18G120200	Glyma.13G346700	Glyma.17G076100
Glyma.15G143600	Glyma.19G221800	Glyma.15G143600	Glyma.19G076200	Glyma.16G119200	Glyma.18G120200
Glyma.16G119200	Glyma.20G035400	Glyma.16G119200	Glyma.20G164900	Glyma.16G173000	Glyma.19G076200
Glyma.16G173000	Glyma.20G164900	Glyma.16G173000		Glyma.17G103500	Glyma.19G221800
Glyma.17G103500		Glyma.17G103500		Glyma.17G217000	Glyma.20G164900
Glyma.17G217000		Glyma.17G217000		Glyma.18G120700	
Glyma.18G120700		Glyma.18G120700		Glyma.18G283400	
Glyma.18G283400		Glyma.18G283400		Glyma.20G035400	
Glyma.19G076200		Glyma.19G221800		Glyma.20G164600	
Glyma.20G164600		Glyma.20G035400		Glyma.20G164700	
Glyma.20G164700		Glyma.20G164600			
		Glyma.20G164700			

Lysobacter enzymogenes		***Pseudomonas fluorescens***		***Rhizobium rhizogenes***	
Inducible	Non-inducible	Inducible	Non-inducible	Inducible	Non-inducible
Glyma.01G055200	Glyma.01G160100	Glyma.01G055200	Glyma.07G061600	Glyma.02G007400	Glyma.01G055200
Glyma.02G007400	Glyma.03G254300	Glyma.01G160100	Glyma.08G299700	Glyma.02G042500	Glyma.01G160100
Glyma.02G042500	Glyma.07G061600	Glyma.02G007400	Glyma.08G300300	Glyma.02G113600	Glyma.07G061600
Glyma.02G113600	Glyma.08G299700	Glyma.02G042500	Glyma.09G038500	Glyma.03G254300	Glyma.08G299700
Glyma.05G075000	Glyma.08G300300	Glyma.02G113600	Glyma.12G049200	Glyma.05G075000	Glyma.08G300300
Glyma.08G259200	Glyma.12G049200	Glyma.03G254300	Glyma.15G143600	Glyma.08G259200	Glyma.09G126200
Glyma.09G038500	Glyma.15G206400	Glyma.05G075000	Glyma.15G206400	Glyma.09G038500	Glyma.12G049200
Glyma.09G126200	Glyma.15G206800	Glyma.08G259200	Glyma.15G206800	Glyma.10G227700	Glyma.13G155800
Glyma.10G227700	Glyma.17G076100	Glyma.09G126200	Glyma.16G119200	Glyma.11G124500	Glyma.15G206400
Glyma.11G124500	Glyma.18G120200	Glyma.10G227700	Glyma.17G076100	Glyma.12G156600	Glyma.15G206800
Glyma.12G156600	Glyma.20G035400	Glyma.11G124500	Glyma.18G120200	Glyma.13G346700	Glyma.17G076100
Glyma.13G155800	Glyma.20G164900	Glyma.12G156600	Glyma.18G283400	Glyma.15G015100	Glyma.18G120200
Glyma.13G346700		Glyma.13G155800	Glyma.19G076200	Glyma.15G143600	Glyma.18G283400
Glyma.15G015100		Glyma.13G346700	Glyma.20G164900	Glyma.16G119200	Glyma.19G076200
Glyma.15G143600		Glyma.15G015100		Glyma.16G173000	Glyma.20G164900
Glyma.16G119200		Glyma.16G173000		Glyma.17G103500	
Glyma.16G173000		Glyma.17G103500		Glyma.17G217000	
Glyma.17G103500		Glyma.17G217000		Glyma.18G120700	
Glyma.17G217000		Glyma.18G120700		Glyma.19G221800	
Glyma.18G120700		Glyma.19G221800		Glyma.20G035400	
Glyma.18G283400		Glyma.20G035400		Glyma.20G164600	
Glyma.19G076200		Glyma.20G164600		Glyma.20G164700	
Glyma.19G221800		Glyma.20G164700			
Glyma.20G164600					
Glyma.20G164700					
